# Solar light degradation of organic dye pollutants and preparation of bis(indolyl)methanes using core-shell Fe_3_O_4_@SiO_2_@CuO nanocomposite

**DOI:** 10.3906/kim-2104-43

**Published:** 2021-08-10

**Authors:** Kaveh PARVANAK BOROUJENI, Zeinab TOHIDIYAN, Mina MIRZAEI

**Affiliations:** 1 Department of Chemistry, Shahrekord University, Shahrekord, Iran; 2 Department of Chemistry, Shahrekord Branch, Islamic Azad University, Shahrekord, Iran

**Keywords:** Core-shell Fe_3_O_4_@SiO_2_@CuO nanocomposite, microwave irradiation, solar-light photocatalytic degradation, bis(indolyl)methanes, catalysis

## Abstract

In this research, a new ferromagnetic-recoverable core-shell Fe_3_O_4_@SiO_2_@CuO nanocomposite of a certain size (20–25 nm) has been synthesized based on Cu(II) complex coated on Fe_3_O_4_@SiO_2_ nanoparticles by facile and fast solid state microwave irradiation method. The photocatalytic activity of the nanocomposite was investigated for degradation of methylene blue (MB) and methyl orange (MO) dye pollutants in aqueous media under solar light irradiation. The nanocomposite could destroy these dyes with high efficiency in short time. With comparison of degradation percentages can be concluded that the nanocomposite shows better photocatalytic activity for MB dye (97% in 180 s). Kinetic study revealed higher rate constant for degradation of MB (k= 3.6×10^−3^ s^−1^) with pseudozero-order model. Also, Fe_3_O_4_@SiO_2_@CuO nanocomposite was an efficient magnetically recoverable catalyst for the preparation of bis(indolyl)methanes (BIMs) through the condensation of an aldehyde with 2 equivalents of indole in EtOH/H_2_O as green solvents.

## 1. Introduction

Nowadays, protection of the environment has received considerable attention. Polluter compounds are spread in the environment from various sources. A type of hazardous polluter compounds are organic dyes, generated by industrial processes. The growing amount of different organic dyes in waste water is a worrying issue for human health and live species [1]. Consequently, it is necessary to develop strategies for removing them from environmental systems. Many techniques such as coagulation, flocculation, reverse osmosis, adsorption on the activated carbon, ultrafiltration, and adsorption have been reported as effective ways to remove the toxic dyes from waste water [2,3]. In recent years, photocatalytic degradation reactions have emerged as an alternative approach for the removal of different dye pollutants from waste water [4]. In fact, photocatalytic degradation reactions are oxidation processes which lead to complete degradation of dye pollutants in short times and even at low concentration. Due to versatility and relative simplicity along with high degradation efficiency, much research effort has focused on design and preparation of various photocatalysts [5,6].

Core-shell nanostructures are complex systems that involve the benefits of both core and shell to improve physiochemical properties. The core usually consists of the inexpensive and easily oxidized metals and the shells include somewhat noble metals, metal oxides, available carbon materials, polymers, silica, and so on [7]. Different methods have been explored in the synthesis of core-shell systems, such as hydrothermal, deposition–precipitation, and sonochemical method [8]. Among core-shell nanostructures, core-shell composites with Fe_3_O_4_ at the center have been widely used because of their characteristic properties such as easy separation from reaction vessel via an external magnet and subsequent use in catalytic reactions, chemical stability, nontoxicity, high selectivity and activity performance, and suitable industrial and biomedical applications [9,10]. However, applications of core-shell composites in catalytic field for preparing organic compounds are rare. For instances, dendrimer–encapsulated Cu(II) nanoparticles supported on superparamagnetic Fe_3_O_4_@SiO_2_ nanoparticles and Fe_3_O_4_@SiO_2_ nanoparticles have been applied as catalysts for the preparation of 5-substituted 1H-tetrazoles [11] and the synthesis of tetrahydrobenzo[a]xanthen-11-ones [12], respectively.

**Scheme 1:**
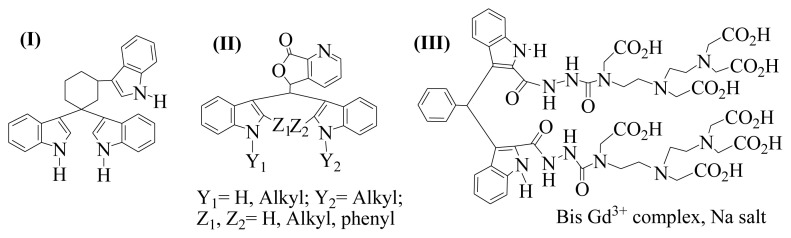
Biological applications of a series of BIM derivatives.

BIMs are compounds consisting of two indolyl moieties which are connected to each other by a carbon atom [13]. BIMs can be obtained either from different earthly and marine natural sources or by the laboratory methods. The prominent biological activities associated with BIMs are antimicrobial, antifungal, analgesic, and antiinflammatory [14,15]. They are inhibitors of bladder cancer [16]. Anticancer effects of 1,1,3-tri(3-indolyl)cyclohexane (Scheme 1, I) in various lung cancer cells were surveyed [17]. Chromogenic 3,3′-bisindolyl-4-azaphthalides (II) [18] has been employed as color formers in pressure-sensitive or heat-sensitive recording materials. Also, complex (III) based on BIMs containing radioactive metal ions (Gd^3+^) [19] has been used as a contrast agent for radio-imaging and visualization of various tissues and organs. One of the most frequently used methods for the preparation of BIMs is the electrophilic substitution reaction of indole with aldehydes and ketones. This reaction is usually catalyzed by several types of catalysts such as InCl_3_ [20], LiClO_4_ [21], I_2_ [22], sulfamic acid [23], HBF_4_–SiO_2_ [24], poly(ethylene-glycol)–sulfonic acid (PEG–SO_3_H) [25], sulfonated polyacrylamide [26], SiO_2_–AlCl_3_ [27], Nafion–H® [28], [DABCO–H][HSO_4_] [29], nano *n*-propylsulfonated γ-Fe_2_O_3_ [30], Sc(OTf)_3_ [31], CaO [32], graphene oxide [33], 4-(3-methylimidazolium)butane sulfonate (MBS) [34], hyper-cross-linked polyaromatic spheres (HCP@CH_2_Br) [35], itaconic acid [36], borophosphate glasses [37], and BF_3_–grafted Fe_3_O_4_@sucrose nanoparticles [38]. However, many of these reported synthetic protocols suffer from some disadvantages like long reaction time, low yields, strongly acidic conditions, tedious work-up, the use of toxic solvents, formation of by-products, and difficulty of recovery and recycling. Therefore, the preparation of BIMs seems still a challenge, especially from the viewpoint of green chemistry.

Recently, we introduced solid-state microwave method as a newer and ‘greener’ synthetic methodology for the preparation of NiO nanoparticles [39]. This method has interesting features and might find application for the preparation of nanomaterials with improved properties which can provide considerable benefit in the fields of medicine. We also used this method for the preparation of Co–Sn–Cu oxides/graphene nanocomposites as green and recyclable catalysts for preparing 1,8-dioxo-octahydroxanthenes and apoptosis-inducing agents in MCF-7 human breast cancer [40]. The superiority of solid-state microwave method over the above mentioned conventional methods [8,41,42] for the synthesis of core-shell systems are reduction of physical and chemical cost, simplicity, safety, generation of pure nanoparticles as well as this method is green (no surfactant) and fast. Along this line, herein, we want to synthesize core-shell Fe_3_O_4_@SiO_2_@CuO nanocomposite by solid-state microwave method based on a novel nano-sized Fe_3_O_4_@SiO_2_@CuL precursor, prepared from the reaction of the Fe_3_O_4_@SiO_2_ with a copper (II) Schiff base complex (CuL). Photocatalytic activity of the synthesized core–shell Fe_3_O_4_@SiO_2_@CuO nanocomposite was investigated for degradation of the cationic and anionic organic dye pollutants (MB and MO, respectively) under solar light. Also, this nanocomposite was applied as a new heterogeneous catalyst for the preparation of BIMs under mild conditions.

## 2. Experimental

### 2.1. General

The reagents and solvents were either prepared in our laboratory or were purchased from commercial suppliers Merck and Fluka. For the microwave irradiation, a microwave oven (LG: MH6535GISW, 1700 W, Korea) was used. Ultrasonic (US) generator was carried out on ultrasonic probe (Top-Sonics UPH-400, Germany). Proton nuclear magnetic resonance (^1^H NMR) spectra were recorded on 400 MHz spectrometer (Bruker, Germany) in deuterated chloroform. Fourier-transformed infrared spectroscopy (FT-IR) was used to obtain spectra of samples using a Schimadzu system FT-IR 8400 spectrophotometer (Japanese) by KBr pellets. X-ray diffraction (XRD) analysis was carried out using a Rigaku D-max C III, X-ray diffractometer with Ni-filtered Cu Ka radiation (PANalytical X’Pert Pro, Netherlands). The morphology of samples was founded with field emission scanning electron microscopy (FESEM) that was taken on a Hitachi s4160/Japan with gold coating that was equipped with a link EDX analyzer. The magnetic property of the sample was measured by a vibrating sample magnetometer (VSM, Meghnatis Kavir Kashan Co., Kashan, Iran) at room temperature. UV–Vis spectra were measured on a double-beam Shimadzu 1650 PC UV–Vis (Japanese) and the samples were dispersed in 20 mL EtOH at room temperature for 20 min. Melting points were found out using a Fisher–Jones melting-point apparatus (USA). Reaction monitoring was accomplished by thin layer chromatography (TLC) (silica gel 60-F250 precoated, England). Transmission electron microscopy (TEM) was imaged by Philips CM30 (Netherlands), a 300kV.

### 2.2. Preparation of the nano-sized Fe_3_O_4_@SiO_2_

Fe_3_O_4_ magnetic nanoparticles were prepared by Fathirad’s method [43]. Firstly, a solution of 20 mL FeSO_4_.2H_2_O (1.6 g, aq.) and 50 mL FeCl_3_.6H_2_O (3.8 g, aq.) was prepared. Then, 10 mL NH_3_ solution (25%) was slowly added to the above solution at the presence of ultrasonic irradiation (100 W) for 15 min under N_2_ atmosphere. After a few minutes, a black suspension was generated and then the Fe_3_O_4_ magnetic nanoparticles were separated using an external permanent magnet. The nanoparticles were washed with distilled water, ethanol, and dried under vacuum at 70 °C. After the preparation of Fe_3_O_4_ nanoparticles, Fe_3_O_4_@SiO_2_ structure was prepared by coating the Fe_3_O_4_ nanoparticles with tetraethyl orthosilicate (TEOS). For this purpose, to 30 mL ethanolic suspension of the obtained Fe_3_O_4_ was slowly added 4 mL TEOS. Then, 12 mL aqueous solution of ammonia (25 %) was added to this solution in the presence of ultrasonic irradiation (100 W) for 10 min. The dark brown precipitate, Fe_3_O_4_@SiO_2_, was formed with stirring at room temperature after 24 h. The obtained Fe_3_O_4_@SiO_2_ was filtered off, washed with methanol, and dried under vacuum at room temperature.

### 2.3. Preparation of 4,4′-dibromo-2,2′-[cyclohexane-1,2-diylbis(nitrilomethanylylidene)]diphenol (H_2_L)

To a 20 mL of methanolic solution of 5-bromosalicylaldehyde (0.4 g, 2 mmol) was added 1,2-diaminocyclohexane (0.1 g, 1 mmol) with continuous stirring. Then, the above solution was refluxed until a yellow precipitate was formed after 60 min. The precipitate was filtered, washed with methanol and ether, and dried under vacuum at 50 °C. The solid crude product was recrystallized from a solution of MeOH and DMF (2:1 in volume) to yield pure crystals of H2L after several days [44].

### 2.4. Preparation of copper (II) Schiff base complex (CuL)

H_2_L ligand (0.3 mmol, 0.15 g), in methanol (10 mL), was exposed to ultrasonic irradiation (150 W). Afterwards, 10 mL methanolic solution of Cu(CH_3_COO)_2_.2H_2_O (0.3 mmol, 0.14 g) was added dropwise to the above mixture, followed by 15 min sonication at room temperature. The mixture was filtered and the CuL powder (brown color) was washed successively with methanol and diethyl ether, and dried in air at ambient temperature overnight [45].

### 2.5. Preparation of the nano-sized Fe_3_O_4_@SiO_2_@CuL precursor

A suspension of Fe_3_O_4_@SiO_2_ (1 g) in chloroform (50 mL) was prepared by sonication and then excess amount of CuL Schiff base complex (1.2 g, 2 mmol) was added dropwise to the prepared suspension under ultrasonic irradiation for 30 min. The obtained dark brown suspension was stirred at room temperature for 12 h, afterwards the Fe_3_O_4_@SiO_2_@CuL product was filtered off, washed twice with chloroform and diethyl ether, and dried under vacuum at room temperature overnight.

### 2.6. Preparation of the core-shell Fe_3_O_4_@SiO_2_@CuO nanocomposite

To prepare core-shell Fe_3_O_4_@SiO_2_@CuO nanocomposite, 2 g of the Fe_3_O_4_@SiO_2_@CuL precursor was poured into a porcelain crucible and it was placed in another bigger porcelain crucible, filled with CuO powder (as microwave irradiation absorber). The collection was placed in a microwave oven under microwaves irradiation in air (950 W, 350 °C). The generated heat led to the decomposition of precursor sample. After 10 min, decomposition of the Fe_3_O_4_@SiO_2_@CuL precursor was completed. The Fe_3_O_4_@SiO_2_@CuO nanocomposite product was washed with ethanol and dried under vacuum at room temperature overnight.

### 2.7. Photocatalytic tests

The photocatalytic ability of the core-shell Fe_3_O_4_@SiO_2_@CuO nanocomposite was surveyed for the removal of MB or MO as organic dye pollutants from aqueous solutions. Firstly, the dosage of photocatalyst (0.004–0.007 mg), amount of H_2_O_2_ (0–5 mL), and pH of the solution (5–10) were selected and optimized. The photocatalytic tests were performed on the days (10 AM to 2 PM) of bright sunny light (average light intensity of 180 mW cm^−2^). Typically, 0.005 g of the Fe_3_O_4_@SiO_2_@CuO nanocomposite as photocatalyst was added to 50 mL of MB or MO aqueous solution with concentration of 4 ppm and the mixture was stirred (500 rpm) at room temperature in dark for 30 min, in order to establish an adsorption–desorption equilibrium between catalyst and dye. Then, the mixture was exposed to solar light in the presence of an appropriate amount of H_2_O_2_ (30%) at suitable pH, and consequently the degradation process of the dye took place. The degradation of MB and MO dyes was carried out at pH = 7 using 2 mL H_2_O_2_ and at pH = 9 using 1 mL H_2_O_2_, respectively. At determined time intervals, the photocatalyst powder was isolated by centrifugation of 3 mL of the mixture and the degradation process of each dye was estimated by measuring the absorptions of MB and MO dyes at 663 and 462 nm, respectively, on their corresponding UV–Vis spectra. The pH of solution was adjusted to determined values by the dropwise addition of HCl (1 M) or NaOH (1 M) to the solution. The percent of degradation was measured by the following equation (Eq. (1)):


$$Degradation\;(\%)=\frac{C0-Ct}{C0}\times 100,$$

where C_t_ is the final concentration of dye solution after a determined time (t) and C_o_ is the initial concentration of dye solution. After each experiment, the Fe_3_O_4_@SiO_2_@CuO nanocomposite was separated from the solutions by an external permanent magnet and then it was washed several times with water and ethanol and dried at 70 °C, and reused for the next experiments.

### 2.8. Typical procedure for the preparation of BIMs

A mixture of 2-thienyl carbaldehyde (1 mmol) and indole (2 mmol) was placed in a round-bottom flask containing 5 mL of EtOH/H_2_O (1:1). Subsequently, Fe_3_O_4_@SiO_2_@CuO nanocomposite catalyst (0.03 g) was added to the mixture and stirred at 80 °C. After the completion of the reaction as followed by TLC (chloroform), the catalyst was separated by an external permanent magnet. The remaining mixture was concentrated on a rotary evaporator under reduced pressure to give the desired product. The products were purified by recrystallization from ethanol or chromatographed on silica plates with chloroform as eluent where necessary.

## 3. Results and discussion

### 3.1. Synthesis and structural characterization of nanocomposites

**Scheme 2:**
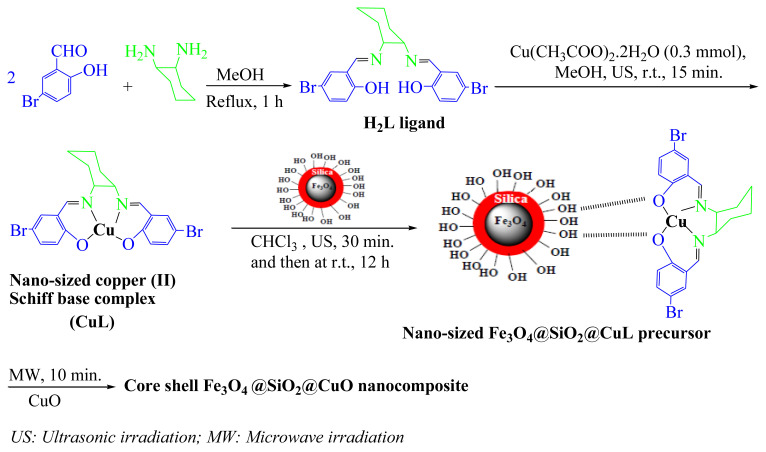
The synthetic routes for Fe_3_O_4_@SiO_2_@CuO nanocomposite.

The core-shell Fe_3_O_4_@SiO_2_@CuO nanocomposite was prepared from Fe_3_O_4_@SiO_2_@CuL as a new precursor by solid-state microwave method (Scheme 2). In the first step, Fe_3_O_4_ nanoparticles reacted with TEOS to afford the core-shell Fe_3_O_4_@SiO_2_ [43]. In the second step, the nano-sized new copper (II) Schiff base complex (CuL) was synthesized from the chemical reaction of 1,2-diaminocyclohexane with 5-bromosalicylaldehyde, followed by the reaction with copper salt, Cu(CH_3_COO)_2_.2H_2_O, under ultrasonic irradiation [44,45]. Afterwards, Fe_3_O_4_@SiO_2_@CuL precursor was prepared from the treatment of Fe_3_O_4_@SiO_2_ with CuL Schiff base complex under ultrasonic irradiation. In the next step, Fe_3_O_4_@SiO_2_@CuL precursor was exposed to microwave irradiation in order to be converted to core-shell Fe_3_O_4_@SiO_2_@CuO nanocomposite. However, the precursor remained unchanged for 30 min, which shows the compound cannot absorb microwaves. Therefore, it seems that attendance of microwave absorber is required. For this purpose, CuO powder was used as microwave irradiation absorber. By using CuO, we observed that the precursor was completely decomposed during the absorption of heat from the hot CuO and the nano-sized Fe_3_O_4_@SiO_2_@CuO powder was generated through the equation below (Eq. (2)) [46]. Note that in the synthesized core-shell Fe_3_O_4_@SiO_2_@CuO, Fe_3_O_4_ is a magnetic core which increases magnetic property of particles and SiO_2_ is chosen as an intermediary layer for the connection between shell layer (CuO) and the core (Fe_3_O_4_).


Eq. (2) $${\text{Fe}}_{3}{\text{O}}_{4}@{\text{SiO}}_{2}@\text{CuL}\to {\text{Fe}}_{3}{\text{O}}_{4}@{\text{SiO}}_{2}@\text{CuO}+({\text{N}}_{2}+\text{NO}+{\text{N}}_{2}\text{O})+{\text{H}}_{2}\text{O}$$

**Figure 1:**
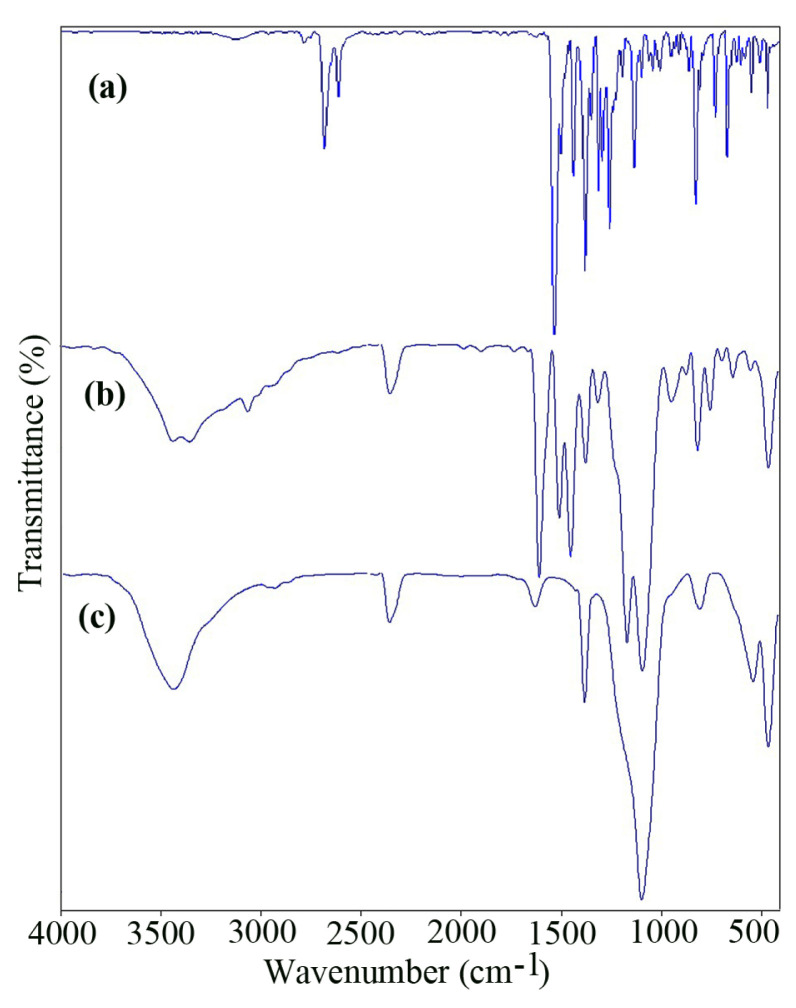
FT-IR spectra of the nano-sized CuL complex (a), Fe_3_O_4_@SiO_2_@CuL precursor (b), and Fe_3_O_4_@SiO_2_@CuO nanocomposite (c).

Figures 1a and 1b show the FT-IR spectra of the nano-sized CuL complex and Fe_3_O_4_@SiO_2_@CuL precursor, respectively. In Figure 1b, the band at 1609 cm^−1^ was attributed to C=N group that show the red shift to lower frequency compared with those of CuL complex (1640 cm^−1^) [45] as a result of the metal complex formation. The peaks at 1095 and 466 cm^−1^ were assigned to Si–O and Fe–O bands, respectively [47]. By comparison of the IR spectrum of the nano-sized Fe_3_O_4_@SiO_2_@CuO (Figure 1c) with Fe_3_O_4_@SiO_2_@CuL precursor (Figure 1b), it will be clear that the bands of CuL complex were diminished and stretching frequencies of Si–O and Fe–O bands shifted toward 1097 and 467 cm^−1^, respectively. In addition, in Figure 1c, the distinct band at 542 cm^−1^ was related to the Cu–O band in monoclinic phase [48] and the bands at 1630 and 3300^−^3400 cm^−1^ could be assigned to the bending vibration and stretching vibration of H_2_O absorbed by KBr pellets or the sample, respectively [45]. Thus, the IR spectral results indicate the successful decomposition of Fe_3_O_4_@SiO_2_@CuL precursor by solid-state microwave method and formation of Fe_3_O_4_@SiO_2_@CuO nanocomposite.

**Figure 2:**
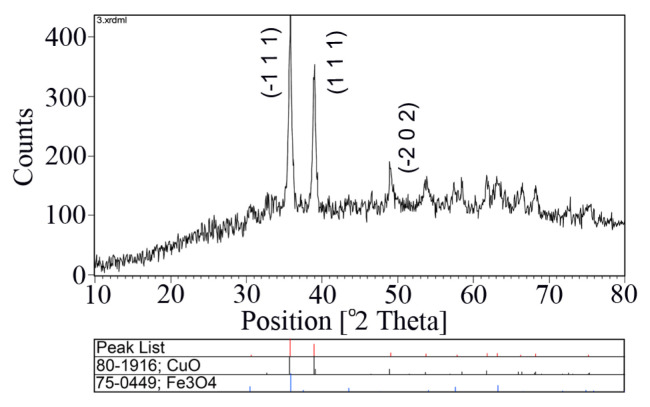
XRD pattern of the Fe_3_O_4_@SiO_2_@CuO nanocomposite.

Figure 2 exhibits XRD analysis of the synthesized Fe_3_O_4_@SiO_2_@CuO nanocomposite. The XRD result manifests the peaks of CuO in monoclinic phase (Space group: Cc, No: 9). The crystallographic parameters of a, b, and c are 4.69, 3.42 and 5.13 Å, respectively. Also, values of α, β, and γ parameters are 90.00°, 99.54°, and 90.00°, respectively. The significant peaks appeared at 2θ = 35.77°, 38.95°, and 49.07° that can be exactly related to (−1 1 1), (1 1 1), and (−2 0 2) planes of crystal, respectively. In this pattern, only CuO (JCPDS Card No. 80–1916) and Fe_3_O_4_ (JCPDS Card No. 75–0449) phases were observed. It is obvious that SiO_2_ have amorphous structure [49]. As shown in Figure 2, there are no peaks of impurity, suggesting that the pure crystalline Fe_3_O_4_@SiO_2_@CuO was formed via solid state decomposition of Fe_3_O_4_@SiO_2_@CuL precursor under microwave irradiation. Also, in the XRD pattern, wide width of the peaks is due to the formation of small size particles of the Fe_3_O_4_@SiO_2_@CuO (Table 1). The mean size of the Fe_3_O_4_@SiO_2_@CuO particles calculated by the Debye–Scherrer equation was found to be 23.83 nm [50].

**Figure 3:**
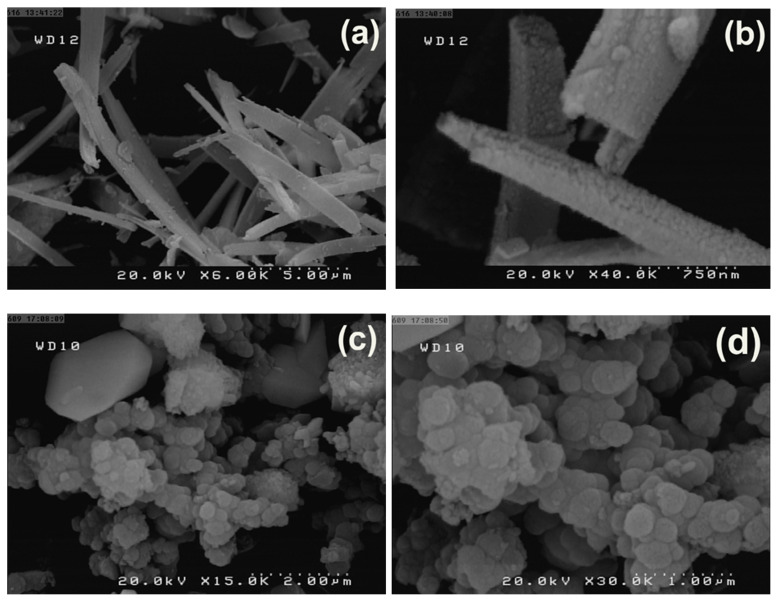
FESEM of the Fe_3_O_4_@SiO_2_@CuL precursor (a,b) and Fe_3_O_4_@SiO_2_@CuO nanocomposite (c,d) at different magnifications.

The morphology of the synthesized Fe_3_O_4_@SiO_2_@CuL precursor and Fe_3_O_4_@SiO_2_@CuO nanocomposite were investigated by FESEM analysis. The images of the Fe_3_O_4_@SiO_2_@CuL precursor were shown in Figures 3a and 3b at different magnification. As it can be seen, the morphology of the precursor is nanorod. However, from Figures 3c and 3d, it is clear that the morphology of Fe_3_O_4_@SiO_2_@CuO particles are spherical shape and they are quite different from that of the precursor compound. Therefore, these results reveal that Fe_3_O_4_@SiO_2_@CuL precursor was converted to Fe_3_O_4_@SiO_2_@CuO particles by solid-state microwave method.

**Figure 4:**
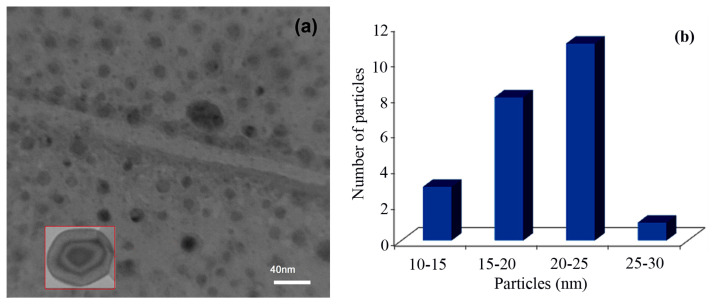
TEM image of core-shell Fe_3_O_4_@SiO_2_@CuO nanocomposite (a) and particle size distribution (b).

Figure 4a displays TEM analysis of the Fe_3_O_4_@SiO_2_@CuO nanocomposite. A typical image of the synthesized sample shows core-shell shape of uniform nano crystalline structures. The black spot shows Fe_3_O_4_ core which is surrounded by SiO_2_. Also, ashen parts after SiO_2_ regions illustrate CuO shell. The particle size distribution of nanocomposite is 20–25 nm (Figure 4b). The particle size estimated by XRD diffraction pattern and the TEM analysis have good match with each other.

**Figure 5:**
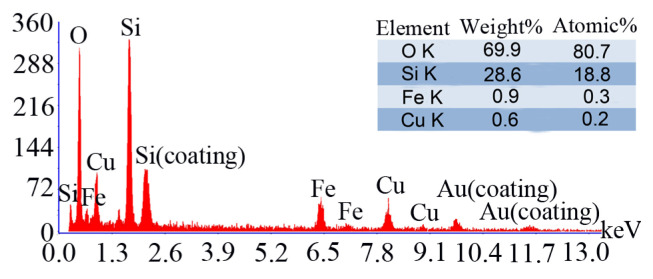
EDX spectrum of the Fe_3_O_4_@SiO_2_@CuO nanocomposite.

EDX spectrum of the core-shell Fe_3_O_4_@SiO_2_@CuO nanocomposite is illustrated in Figure 5 and contains signals of Cu, Fe, Si, and, O elements. The Au and Si signals (notated as coating) were observed due to the instrument. Additionally, the weight and atomic percentages of the resided elements in obtained nanocomposite have been shown in a table inserted in Figure 5. Therefore, from the above results, it can again be concluded that Fe_3_O_4_@SiO_2_@CuO nanocomposite was papered by solid-state microwaved method.

**Figure 6:**
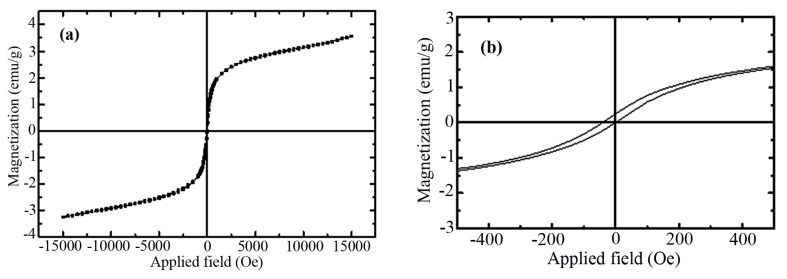
Magnetization versus applied magnetic field for Fe_3_O_4_@SiO_2_@CuO nanocomposite at room temperature (a) and enlarged view of the hysteresis loop in the low-field region (b).

The alteration in magnetization (M) vs. applied field (H) for the core-shell Fe_3_O_4_@SiO_2_@CuO nanocomposite at room temperature with field sweeping from −15,000 to +15,000 Oe is shown in Figure 6. The hysteresis loop shows a weak ferromagnetic behavior. The hysteresis loop of nano-sized materials was related to the magnetic anisotropy of the lattice, domain structure (pinning effect of magnetic domain walls at grain boundaries), as well as impurities within the nano-sized structures [51]. The remnant magnetization (Mr) and saturation magnetization (Ms) were found to be 0.25 and 3.22 emu g^−1^, respectively. The value of coercive field (Hc) was estimated as 0.053 Oe. The magnetic property of synthesized core-shell Fe_3_O_4_@SiO_2_@CuO nanocomposite may be attributed to the different parameters such as sample shape, size, crystallinity, magnetization direction, and synthetic method.

**Figure 7:**
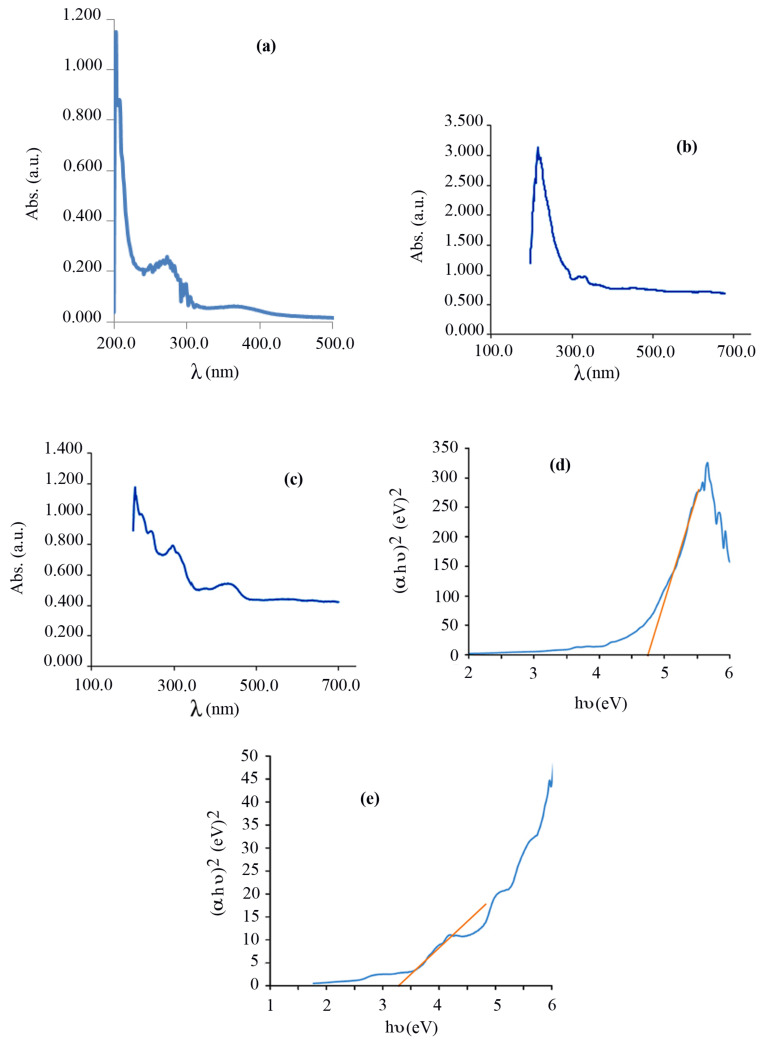
Electronic spectra of Schiff base complex CuL (a), the Fe_3_O_4_@SiO_2_@CuL precursor (b), and core-shell Fe_3_O_4_@SiO_2_@CuO nanocomposite (c) and (αhυ)^2^–hυ curves of the Fe_3_O_4_@SiO_2_@CuL precursor (d) and core-shell Fe_3_O_4_@SiO_2_@CuO nanocomposites (e).

The optical property of the prepared Schiff base complex CuL, nano-sized Fe_3_O_4_@SiO_2_@CuL precursor, and core-shell Fe_3_O_4_@SiO_2_@CuO nanocomposite was studied by UV–Vis spectroscopy. Figure 7a indicates the absorbance spectrum of the Schiff base complex CuL. The bands at 200–300 nm were due to π–π* and n–π* transitions. The band at around 380 nm was related to ^2^B1g → ^2^Eg transition at D_4_h field [52]. In the Fe_3_O_4_@SiO_2_@CuL spectrum (Figure 7b), the strong absorption band was observed at around 250 nm, which is due to the π-π* transitions of the phenolic rings [53]. Also, the bands appeared at 300–400 nm were related to the charge transfer transitions, MLCT and LMCT (metal ligand charge transfer and ligand metal charge transfer, respectively), which have shifted in comparison with those of CuL complex [45], due to the formation of Fe_3_O_4_@SiO_2_@CuL. In Figure 7c, the bands observed are related to the electronic transition from Cu (3d) to O (2p) orbitals [54].

The band gap of the semiconductors can be calculated by Tauc’s equation (Eq. (3)):


$${\left(\alpha \text{h}\upsilon \right)}^{1/\text{n}}=\text{A}(\text{h}\upsilon -E_{\text{g}}),$$

where α, hυ, A, and *E*_g_ are coefficient of absorption (cm^−1^), energy of photon (eV), proportionality constant, and the band gap energy (eV), respectively. The value of the exponent denotes the nature of the electronic transition (allowed or forbidden), and whether it is direct or indirect: n =1/2 for direct allowed transitions, n = 3/2 for direct forbidden transitions, n = 2 for indirect allowed transitions, and n = 3 for indirect forbidden transitions. Plotting the (αhυ)^1/n^ versus (hυ) is a matter of testing n =1/2 or n = 2 to compare which provides the better fit and thus identifies the correct transition type. Figures 7d and 7e show curves of (ahυ)^2^-hυ for the Fe_3_O_4_@SiO_2_@CuL precursor and core-shell Fe_3_O_4_@SiO_2_@CuO nanocomposite, respectively, showing a direct allowed transition. The linear region has been used to extrapolate to the X-axis intercept to find the *E_g_* value. Using this concept, the *E_g_* values of Fe_3_O_4_@SiO_2_@CuL precursor and core-shell Fe_3_O_4_@SiO_2_@CuO nanocomposite were found to be 4.7 and 3.2 eV, respectively. *E_g_* values of Fe_3_O_4_ and Fe_3_O_4_@SiO_2_ have been found to be 1.3 and 1.68 eV, respectively [55]. As it can be seen, *E_g_* of core-shell Fe_3_O_4_@SiO_2_@CuO nanocomposite has shifted in comparison with those of Fe_3_O_4_@SiO_2_@CuL precursor. The band of core-shell Fe_3_O_4_@SiO_2_@CuO nanocomposite shows red shift toward CuO thin films [56] or blue shift in comparison with quantum dots of CuO [57]. This difference is probably related to morphology, size, and effect of the present elements or synthetic method.

**Figure 8:**
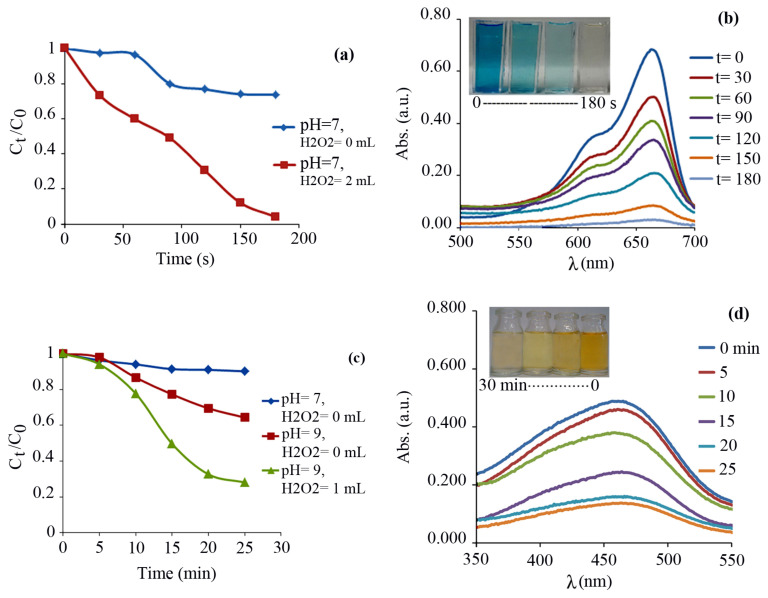
Concentration changes (C_t_/C_0_) versus irradiation time and time-dependent absorption spectrum during degradation process of a 4 ppm aqueous solution of MB (a,b, respectively) and MO (c,d, respectively) dyes in the presence of the core-shell Fe_3_O_4_@SiO_2_@CuO nanocomposite (0.005 g) as a photocatalyst.

Because of a moderate band gap (3.2 eV) of core-shell Fe_3_O_4_@SiO_2_@CuO nanocomposite, we anticipated that it can act as a photocatalyst for destroying the dye pollutants. For this purpose, the photocatalytic activity of core-shell Fe_3_O_4_@SiO_2_@CuO nanocomposite was investigated for solar light degradation of MB and MO organic dye pollutants. Firstly, the experiments were carried out for degradation of MB as a typical cationic dye. The degradation of MB dye was investigated at 663 nm at which the dye shows a strong absorption. Figure 8a shows the values of C_t_/C_0_ of MB dye in different conditions. Clearly, the C_t_/C_0_ values under the optimized conditions (pH= 7 and 2 mL of H_2_O_2_) strongly decreased at room temperature duo to the solar light degradation process. The H_2_O_2_ acts as an assisted-degradation and produces more free radicals (HO•), which leads to faster and more effective degradation. However, use of further amounts of hydrogen peroxide, up to critical concentration, will not enhance the rate of dye degradation process [58].

The characteristic absorption bonds of MB dye at optimized conditions at 663 nm at different times are given in Figure 8b. Clearly, the characteristic absorption decreases with the passage of time. The absorption of MB is about zero after 180 s solar light irradiation. During the degradation process, the intense blue color of the initial solution was decreased until it becomes almost colorless, indicating the successful solar light degradation process of MB. It is worth noting that the absorption bands of MB were not shifted at 663 nm, denoting that the degradation of MB is due to the degradation of the chromophore groups [59]. Thus, the core-shell Fe_3_O_4_@SiO_2_@CuO nanocomposite is an efficacious photocatalyst for the degradation of MB dye in short time with a degradation efficiency of 97 %. In order to demonstrate the effectiveness of photons in dye degradation processes, typically, 0.005 g of the Fe_3_O_4_@SiO_2_@CuO nanocomposite was added to 50 mL of MB or MO aqueous solution with concentration of 4 ppm and the mixture was stirred (500 rpm) at room temperature in dark for 30 min. UV-Vis spectra showed that the significant absorption peaks of dyes were observed without decreasing. Therefore, dye degradation is accomplished in the presence of light.

Based on this encouraging result, the core-shell Fe_3_O_4_@SiO_2_@CuO nanocomposite was subsequently extended to the degradation of MO as a typical anionic dye in similar conditions. The experimental tests showed that the values of C_t_/C_0_ of MO dye under optimized conditions (pH = 9 and 1 mL of H_2_O_2_) decreased sharply at room temperature (Figure 8c). The intensity change of the absorption bands at 462 nm over of the Fe_3_O_4_@SiO_2_@CuO nanocomposite catalyst is plotted in Figure 8d as a function of time. After 25 min, the degradation efficiency for MO was estimated as 72 %. When the results obtained for the degradation of MB and MO dyes using the Fe_3_O_4_@SiO_2_@CuO nanocomposite were compared with each other, it was revealed that the degradation of MB dye (the cationic dye) was performed with higher yield in a shorter time, which indicates that the surface of the Fe_3_O_4_@SiO_2_@CuO nanocomposite catalyst is presumably negatively charged.

**Figure 9:**
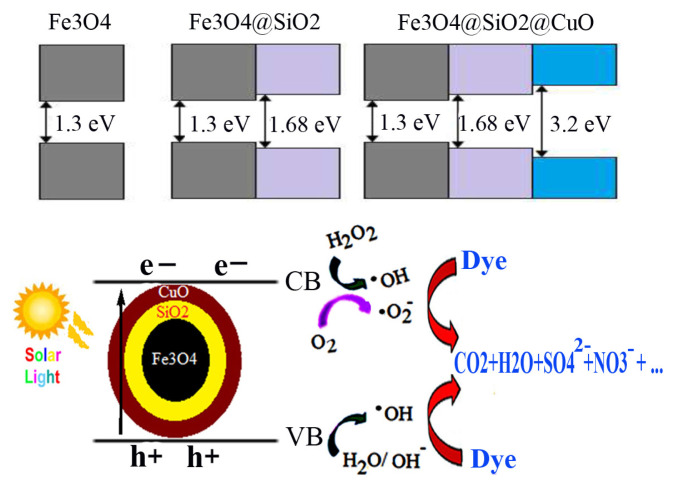
A proposed mechanism of solar light degradation of MB or MO dyes in aqueous solution over the core-shell Fe_3_O_4_@SiO_2_@CuO nanocomposite/H_2_O_2_ system with an energy level diagram. CB and VB are the conduction and the valence bands, respectively.

A radical mechanism for the solar light degradation of MB or MO dyes over core-shell Fe_3_O_4_@SiO_2_@CuO nanocomposite is proposed in Figure 9 [55]. During irradiation, Fe_3_O_4_@SiO_2_@CuO nanocomposite (*E_g_*= 3.2 eV) can create electron-hole pairs (Eq. (4)). The electrons on the conductive band (CB) react with H_2_O_2_ molecules to produce HO• and •O_2_^−^ radicals (Eqs. 5 and 6). Meanwhile, holes on the valence band (VB) would be reacted with the H_2_O molecules or OH^−^ ions to form HO• radicals (Eq. (7)). The produced active HO• and •O_2_^−^ radicals effectively degrade the dye molecules to CO_2_, H_2_O and other inorganic products (Eqs. (8) and (9)).


Eq. (4) $${\text{Fe}}_{3}{\text{O}}_{4}@{\text{SiO}}_{2}@\text{CuO}+\text{h}\upsilon \to {{\text{h}}^{+}}_{\text{VB}}+{{\text{e}}^{-}}_{\text{CB}}$$

Eq. (5) $${{\text{e}}^{-}}_{\text{CB}}+{\text{H}}_{2}{\text{O}}_{2}\to \text{HO}•+{\text{OH}}^{-}$$

Eq. (6) $${{\text{e}}^{-}}_{\text{CB}}+{\text{O}}_{2}\to •{{\text{O}}_{2}}^{-}$$

Eq. (7) $${{\text{h}}^{+}}_{\text{VB}}+{\text{H}}_{2}\text{O}/{\text{OH}}^{-}\to \text{HO}•+{\text{H}}^{+}$$

Eq. (8) $$\text{Dye}+\text{HO}•\to {\text{CO}}_{2},{{\text{NO}}_{3}}^{-},{{\text{SO}}_{4}}^{2-},{\text{H}}_{2}\text{O},\ldots$$

Eq. (9) $$\text{Dye}+•{{\text{O}}_{2}}^{-}\to {\text{CO}}_{2},{{\text{NO}}_{3}}^{-},{{\text{SO}}_{4}}^{2-},{\text{H}}_{2}\text{O},\ldots$$

**Figure 10:**
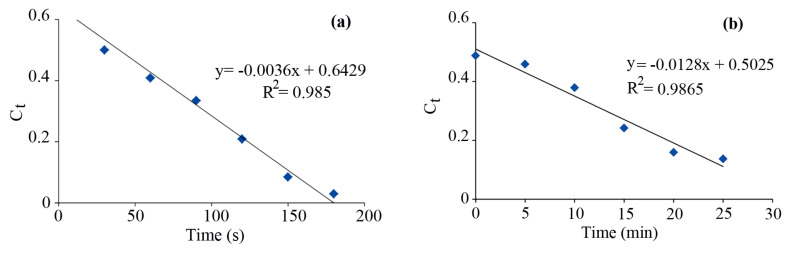
Plot of C_t_ versus irradiation time for MB (a) and MO (b) dyes over Fe_3_O_4_@SiO_2_@CuO nanocomposite. The removal of dyes by this nanocomposite followed pseudo-zero order kinetics.

The solar light degradation kinetics of MB (Figure 10a) and MO (Figure 10b) dyes were determined. The pseudo-zero order model was used: C_t_= −k_t_ + C_0_, where C_0_ and C_t_ are the dye concentrations before and after solar light irradiation, respectively, t is the reaction time as well as k is the rate constant. As shown in Figure 10, the k values for the degradation of MB and MO dyes were found to be 3.6 × 10^−3^ s^−1^ and 0.213 × 10^−3^ s^−1^, respectively. The results indicate that the MB degradation rate is more than those of MO dye. The k value for the photodegradation of MB dye using the Fe_3_O_4_ nanoparticles has been found to be 1.7 × 10^−4^ s^−1^ [60], lower than that of Fe_3_O_4_@SiO_2_@CuO nanocomposite (3.6 × 10^−3^ s^−1^), thus, the latter ones seem to be more effective for dye degradation process.

**Figure 11:**
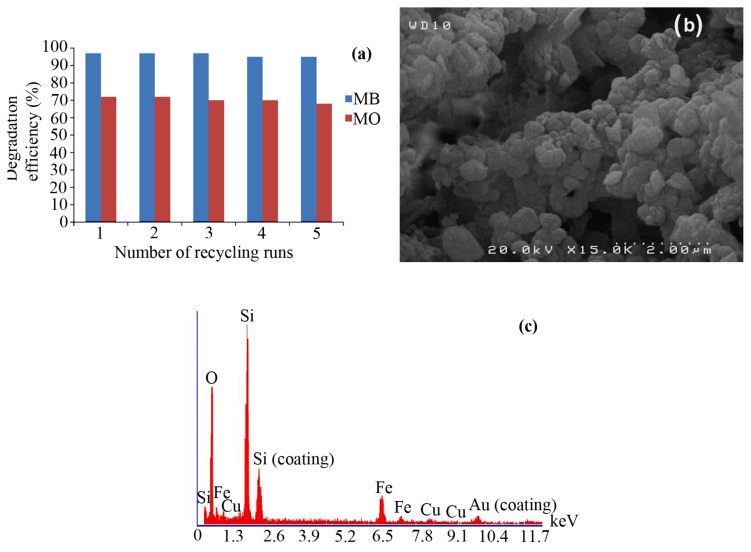
Reusability of core-shell Fe_3_O_4_@SiO_2_@CuO nanocomposite (0.005 g) in the degradation of a 4 ppm aqueous solution of MB and MO dyes after 3 and 25 min, respectively, (a), its FESEM image (b) and EDX spectrum (c) after fifth reuse.

One of the important issues in the catalytic experiments is stability and reusability of the catalysts. The synthesized core-shell Fe_3_O_4_@SiO_2_@CuO nanocomposite is a magnetic material and after each reaction it was easily segregated using an external permanent magnet. After washing core-shell Fe_3_O_4_@SiO_2_@CuO nanocomposite severally with deionized water, the solid was dried and reused for consecutive cycles for the degradation of MB or MB dyes. The results showed that Fe_3_O_4_@SiO_2_@CuO nanocomposite can be reused up to five cycles with no significant decline in degradation efficiency (Figure 11a). Negligible loss in its activity is due to loss of catalyst during separation or through washing cycles. These results were authenticated with FESEM and EDX spectra of Fe_3_O_4_@SiO_2_@CuO nanocomposite after fifth reuse (Figures 11b and 11c), indicating that the structure of the catalyst was preserved after recovery.

Finally, the photocatalytic performance of the synthesized core-shell Fe_3_O_4_@SiO_2_@CuO nanocomposite, for degradation of MB and MO dye pollutants was compared with previously reported literature (Table 2). The results showed that Fe_3_O_4_@SiO_2_@CuO nanocomposite provides better catalytic performances than the previously reported photocatalysts from view of the degradation efficiency and reaction rate. Notably, whereas many catalysts used for the degradation of dyes (entries 1–6) require an additional energy (UV irradiation, ultrasound or microwave source) or heating conditions, Fe_3_O_4_@SiO_2_@CuO nanocomposite does not need an external energy and the degradation process of dyes was carried out only under solar energy. Noteworthy is that in a recent work [66], nanoplate of mixed-Fe_3_O_4_@SiO_2_@CuO synthesized by precipitation method was reported as a photocatalyst for destroying the MO dye. However, the degradation efficiency was low (17.6%) and the degradation process could be accomplished only under UV–Vis irradiation. The superiority of our core-shell Fe_3_O_4_@SiO_2_@CuO nanocomposite over nanoplate of mixed-Fe_3_O_4_@SiO_2_@CuO seems to depend on the differences in morphology and type of precursor, and also applied synthetic method. In the solid-state microwave decomposition method, at the molecular level, microwaves interact with the reactants and the electromagnetic energy is generated. This energy change to heat by rapid kinetics of the molecules and can improve the chemical reaction [39].

**Figure 12:**
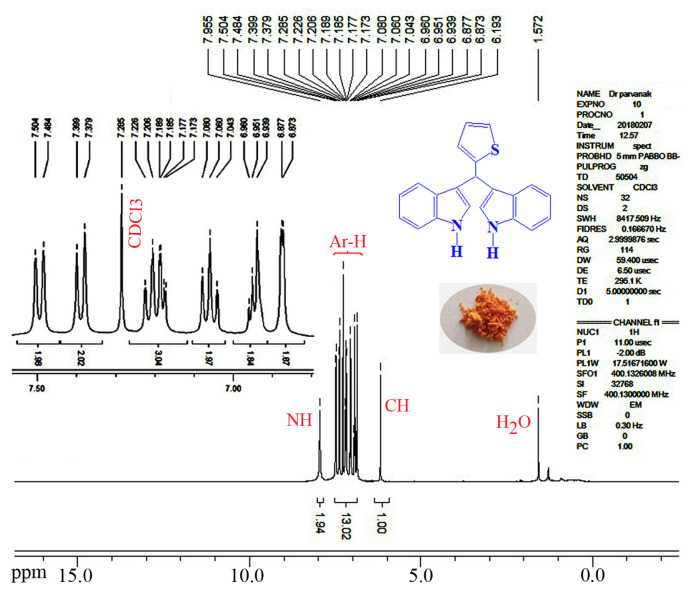
^1^H NMR spectrum of the 3,3′-(2-thienylmethylene)bis-1H-indole in CDCl_3_ as solvent.

Encouraged by the results obtained from the photocatalytic efficiency of Fe_3_O_4_@SiO_2_@CuO in degradation of organic dye pollutants, we turned our attention to examine the catalytic activity of this nanocomposite for the preparation of BIMs. Catalytic experiments were initiated with the reaction of 2-thienyl carbaldehyde (1 equiv.) with indole (2 equiv.) as a typical reaction. To find the optimum reaction conditions, the influence of the solvent nature, reaction temperature, and amount of catalyst were investigated. The best yields were obtained in EtOH/H_2_O (1:1) at 80 °C in the presence of 0.03 g of Fe_3_O_4_@SiO_2_@CuO nanocomposite. After this, the performance of this approach was explored for the preparation of a wide variety of BIMs. BIMs were obtained in excellent yields using Fe_3_O_4_@SiO_2_@CuO nanocomposite from various aliphatic and aromatic aldehydes, having different substituents, and indole (Table 3, entries 1–7, 10–12). Also, heteroaromatic aldehydes smoothly reacted with indole using Fe_3_O_4_@SiO_2_@CuO nanocomposite to yield their corresponding BIMs (entries 8,9). Reaction of ketones with indole was very slow (entry 13) due to steric effects, and the product was afforded only in trace amounts even after extended time (120 min). In all cases, the reaction proceeds smoothly without the formation of any undesirable products, which normally are observed under the influence of strong acid catalysts. The work-up is reduced to a mere separation of the magnetic catalyst and evaporation of the solvent. Figure 12 shows the ^1^H NMR spectrum of the 3,3′-(2-thienylmethylene)bis-1H-indole (as a brick red powder).

**Scheme 3:**
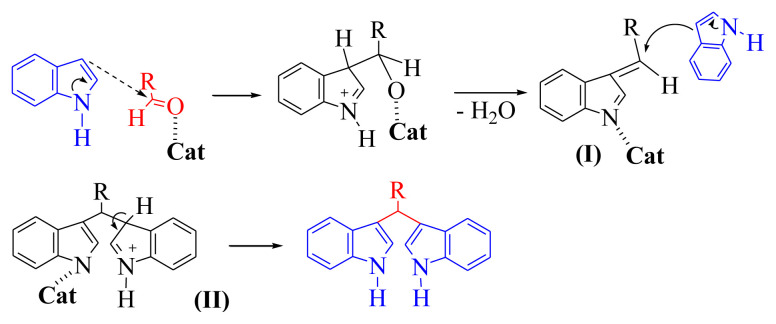
A probable mechanism of the reaction of aldehydes with indole in the presence of Fe_3_O_4_@SiO_2_@CuO nanocomposite as catalyst.

To explore the actual role of Fe_3_O_4_@SiO_2_@CuO nanocomposite in the synthesis of BIMs, we explain a plausible mechanism of the reaction in Scheme 3. An activated aldehyde carry out an electrophilic substitution reaction at C-3 of an indole, which after loss of water yields intermediate I. Addition of another molecule of indole to intermediate I, like the Michael addition fashion, yields intermediate II, which produces the target product after aromatization takes place via deprotonation.

**Figure 13:**
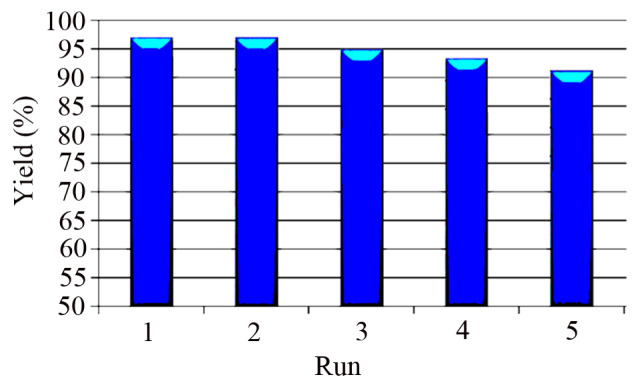
Recyclability of Fe_3_O_4_@SiO_2_@CuO nanocomposite (0.03 g) in the reaction of 2-thienyl carbaldehyde (1 mmol) with indole (2 mmol) in EtOH/H_2_O (1:1) at 80 °C after 20 min.

The reusability of the Fe_3_O_4_@SiO_2_@CuO nanocomposite catalyst in the synthesis of BIMs was studied. The catalyst was reused up to five times without remarkable loss of its efficiency (Figure 13).

A comparison of the present procedure, using Fe_3_O_4_@SiO_2_@CuO nanocomposite, with selected previously reported catalysts is presented in Table 4. Clearly, Fe_3_O_4_@SiO_2_@CuO nanocomposite in addition to having the advantages such as easy separation and recyclability has fine catalytic performance compared to other reported protocols.

## 4. Conclusion

In summary, well-defined core-shell Fe_3_O_4_@SiO_2_@CuO composite was prepared via a fast and efficient solid state microwave irradiation. The composite is a ferromagnetic material in the nano scale range of size (20–25 nm) with a moderate band gap of 3.2 eV, which makes it suitable for applications in areas such as electronics and photonics. The MB and MO dyes were degraded over Fe_3_O_4_@SiO_2_@CuO nanocomposite as a reusable photocatalyst with 97% and 72% efficiency, respectively. The important feature of the present protocol was the use of solar energy to accomplish the degradation of dye pollutants. Also, the nanocomposite exhibited high catalytic activity in the BIMs synthesis. The catalyst is easily separable by an external magnet and its catalytic activity remains after several reaction cycles. The cleaner reaction profiles, simple work-up, no competitive side reactions, high reaction rates, and high yields of the desired products are other advantages of this method. The synthesis and applications of other core-shell nanocomposites with different magnetic cores using solid state microwave method is under investigation.

## Figures and Tables

**Table 1:** XRD data for the Fe_3_O_4_@SiO_2_@CuO nanocomposite.

No.	1	2	3
POS [°2TH]	35.77	38.95	49.07
FWHM [°2TH]	0.38	0.32	0.38
hkl plane	(−1 1 1)	(1 1 1)	(−2 0 2)
Particle size (nm)	21.94	26.33	23.23

**Table 2:** Comparing the photocatalytic efficiency of core-shell Fe_3_O_4_@SiO_2_@CuO nanocomposite with some previously reports in degradation of organic dye pollutants.

No.	Nanomaterial	Dye	Irradiation	Degradation efficiency (%)	Time (min)	Synthetic method
1	CuO	MO	UV	12	120	Precipitation [61]
2	Fe_3_O_4_@CuO-RGO	MB	UV	94	150	Hydrothermal [62]
3	Mn_3_O_4_	MB	Heat	82	24	Sol-gel [63]
4	ZnFe_2_O_4_	MB	MW^a^	32	30	MW^a^ sintering [64]
5	MIL–101(Cr)/RGO^b^/ZnFe_2_O_4_	MB	US^c^	96	50	Hydrothermal [65]
6	MIL-101(Cr)/RGO^b^/ZnFe_2_O_4_	MO	US^c^	80	70	Hydrothermal [65]
7	Core-shell Fe_3_O_4_@SiO_2_@CuO	MB	Solar light	97	3	MW^a^ decomposition
8	Core-shell Fe_3_O_4_@SiO_2_@CuO	MO	Solar light	72	25	MW^a^ decomposition

a Microwave,

b reduced graphene oxide,

c ultrasound.

**Table 3:** Preparation of BIMs.

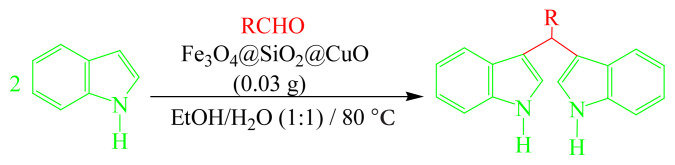				
No.	R	Time (min)	Yield (%)^a^	M.p (°C) (Lit. [Ref])^.^
1	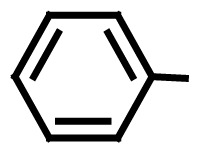	20	96	123–127 (126–127) [23]
2	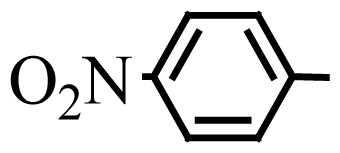	11	97	216–220 (218–220) [36]
3	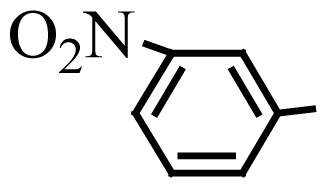	12	95	214–218 (220–222) [36]
4	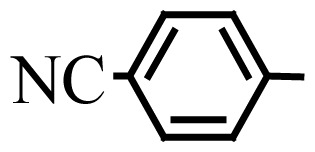	12	94	211–215 (209–211) [23]
5	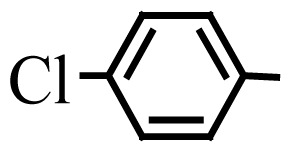	18	96	78–82 (77–78) [29]
6	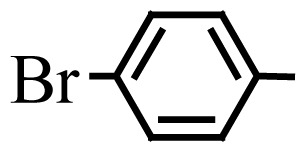	20	95	113–117 (112–113) [23]
7	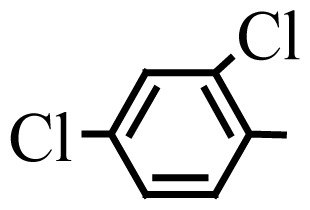	16	96	105–110 (100–102) [29]
8	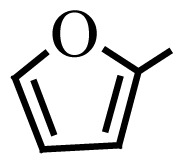	22	93	315–318 (321–322) [36]
9	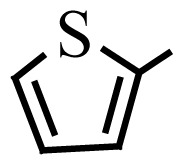	20	97	147–150 (151–153) [22]
10	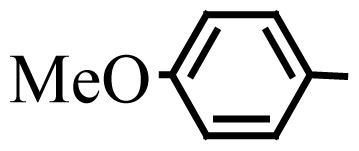	28	93	185–187 (187–189) [22]
11	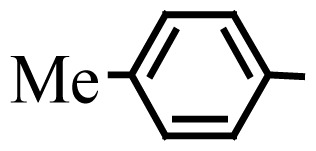	25	93	93–96 (95–97) [23]
12	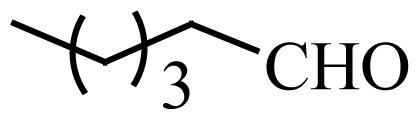	30	92	70–73 (68–70) [21]
13	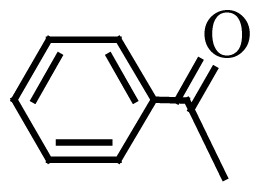	120	trace	-

a Isolated yield. All products are known compounds and were identified by comparison of their physical and spectral data with those of the authentic samples.

**Table 4:** Comparison of the efficiencies of Fe_3_O_4_@SiO_2_@CuO nanocomposite with other reported catalysts for the condensation of indole with benzaldehyde.

No.	Cat.	Solv.	Temp. (°C)	Time (min)	Yield (%)^a^
1	InCl_3_	MeCN	r.t.	240	96 [20]
2	LiClO_4_	MeCN	r.t.	300	90 [21]
3	I_2_	solvent-free	r.t.	10	72 [22]
4	Sulfamic acid	solvent-free	r.t.	92	30 [23]
5	HBF_4_-SiO_2_	solvent-free	r.t.	10	94 [24]
6	PEG–sulfonic acid	MeOH	r.t.	150	95 [25]
7	Sulfonated polyacrylamide	MeCN	reflux	60	95 [26]
8	SiO_2_-AlCl_3_	solvent-free	r.t.	35	96 [27]
9	Nafion-H^®^	PEG/H_2_O	80 °C	25	94 [28]
10	[DABCO-H][HSO_4_]	neat	90 °C	120	91 [29]
11	Nano *n*-propylsulfonated γ-Fe_2_O_3_	solvent-free	80 °C	60	82 [30]
12	Sc(OTf)_3_	THF	r.t.	210	92 [31]
13	CaO	solvent-free	100 °C	210	70 [32]
14	Graphene oxide	H_2_O	40 °C	180	92 [33]
15	MBS	neat	r.t.	60	86 [34]
16	HCP@CH_2_Br	neat	60 °C	60	96 [35]
17	Itaconic acid	H_2_O	100 °C	120	90 [36]
18	Borophosphate glasses	solvent-free	70 °C	90	94 [37]
19	Fe_3_O_4_@sucrose–OBF_3_H	EtOAc	70 °C	150	92 [38]
20	Fe_3_O_4_@SiO_2_@CuO	EtOH/H_2_O	80 °C	20	96

a Isolated yields.
